# Supplementary material for: Toxicity Effects and Mechanisms of MgO Nanoparticles on the Oomycete Pathogen Phytophthora infestans and Its Host Solanum tuberosum
Source: Toxics. 2022 Sep 21;10(10):553. doi: 10.3390/toxics10100553 (PMC9607216; doi:10.3390/toxics10100553)
Supplement: Supplementary file 1 [file toxics-10-00553-s001.zip › toxics-1898476-supplementary/Toxics-SM-Toxicity effects and mechanisms of MgO nanoparticles on the oomycete pathogen Phytophthora infestans and its host Solanum tuberosum.pdf]

toxics

Supplementary Materials: Toxicity effects and mechanisms of MgO nanoparticles on the oomycete pathogen *Phytophthora infestans* and its host *Solanum tuberosum*

Ze-Le Wang, Xi Zhang, Guang-Jin Fan, Yi Que, Feng Xue and Ying-Hong Liu

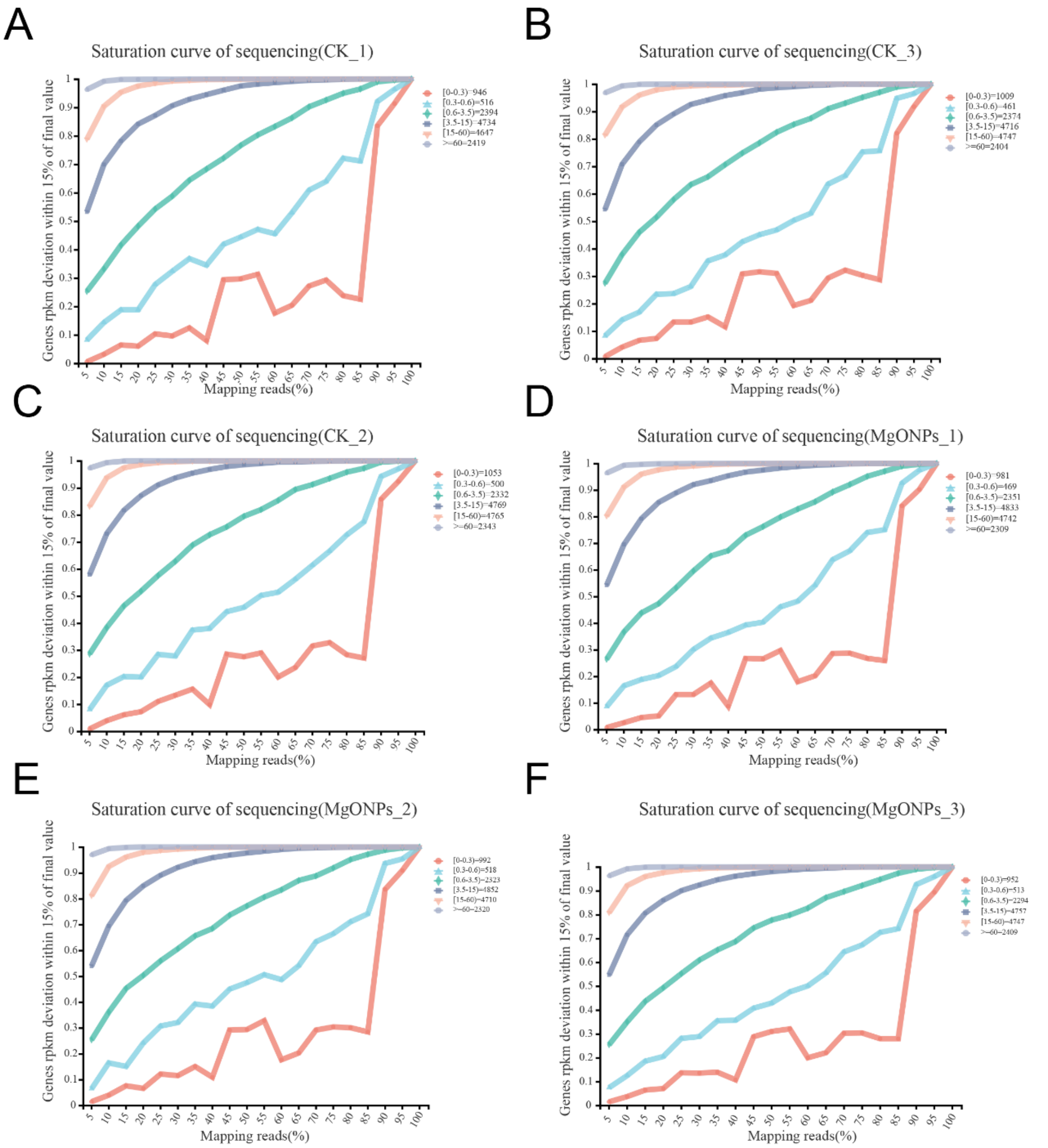

Figure S1. Quantitative saturation curve examination showed requirements of gene expression level for amount of data in each *P. infestans* sample. The X-axis shows percentage of randomly selected reads in total reads. The Y-axis shows the deviation ratio between the calculated expression and the final real expression under current sampling conditions. Each color line represents the different expression level of gene. Higher expression of genes indicates more accurate quantitative measurements. All sequencing data is required for low gene expressions.

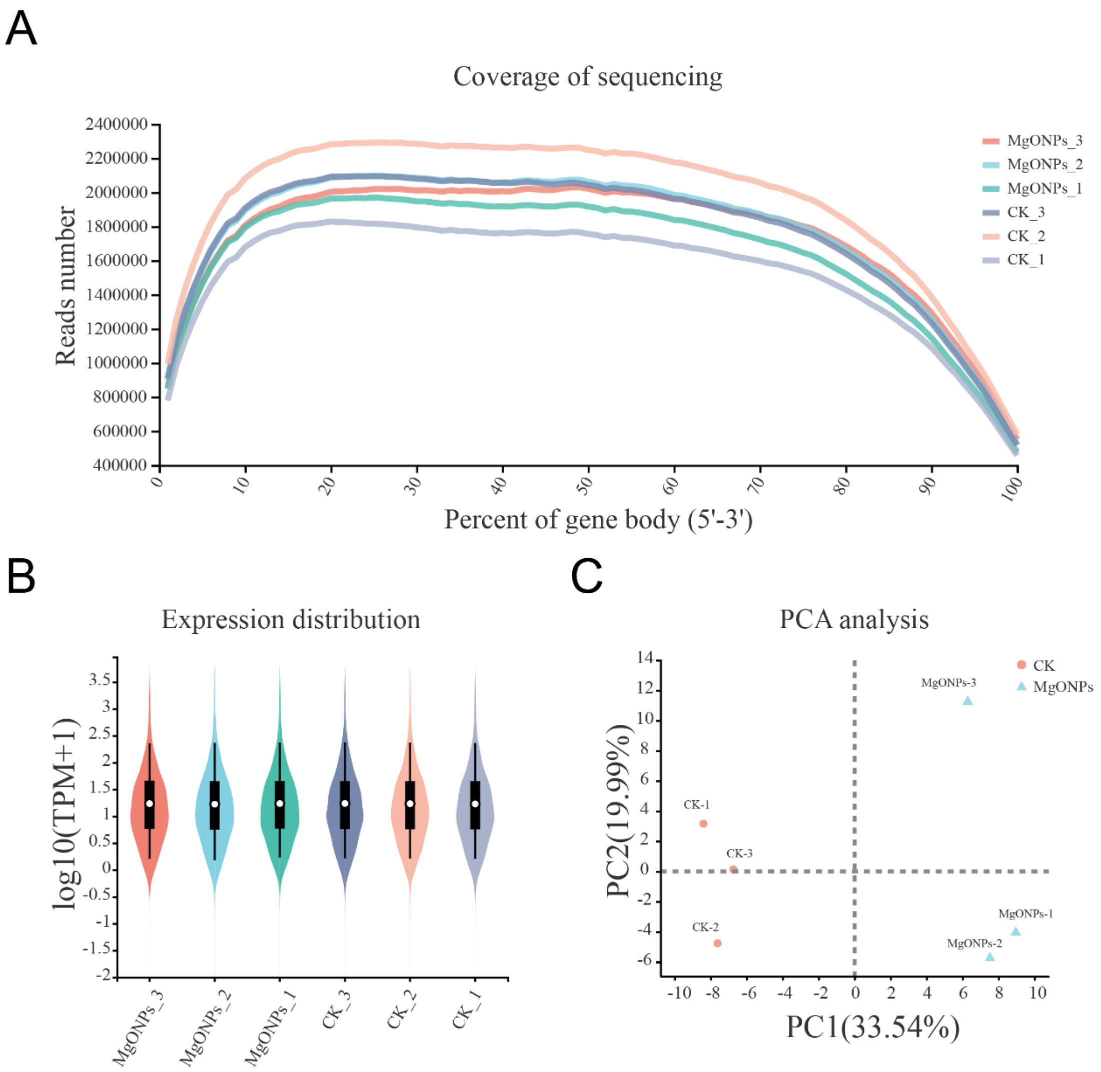

Figure S2. Coverage, expression distribution and principal component analysis (PCA) for different transcriptomes from *P. infestans*. (A) Coverage of sequencing showed that our sequencing has no obvious bias. The X-axis represent the percentage of the base length in total base length of a single gene. "0" represents the 5′ end of the gene, "100" represents the 3′ end of the gene. The Y-axis showed that number of reads which can align to the corresponding gene region on the horizontal axis. The curve has no obvious deviation peak, so our sequencing has no obvious bias. (B) Boxplot showed that the distribution of all TPMs in each sample. The ordinate is log_{10}(TPM+1), each color in the figure represents a sample, and the horizontal line in the figure represents the median level of gene expression in the sample. (C) The correlation analysis between different transcriptomes by principal component analysis (PCA).

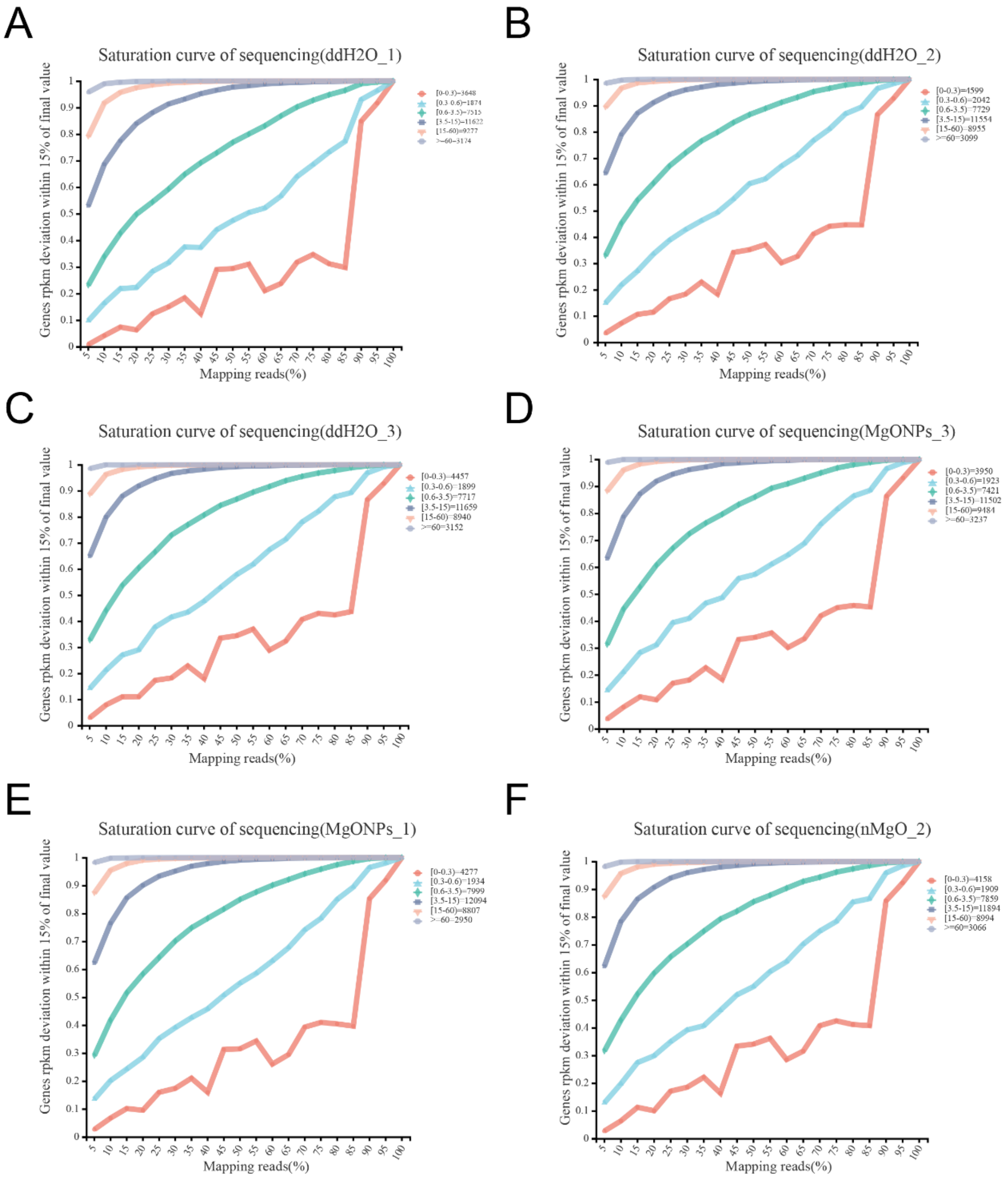

Figure S3. Quantitative saturation curve examination showed requirements of gene expression level for amount of data in each potato sample. The X-axis shows percentage of randomly selected reads in total reads. The Y-axis shows the deviation ratio between the calculated expression and the final real expression under current sampling conditions. Each color line represents the different expression level of gene. Higher expression of genes indicates more accurate quantitative measurements. Large amount of sequencing data is required for low gene expressions.

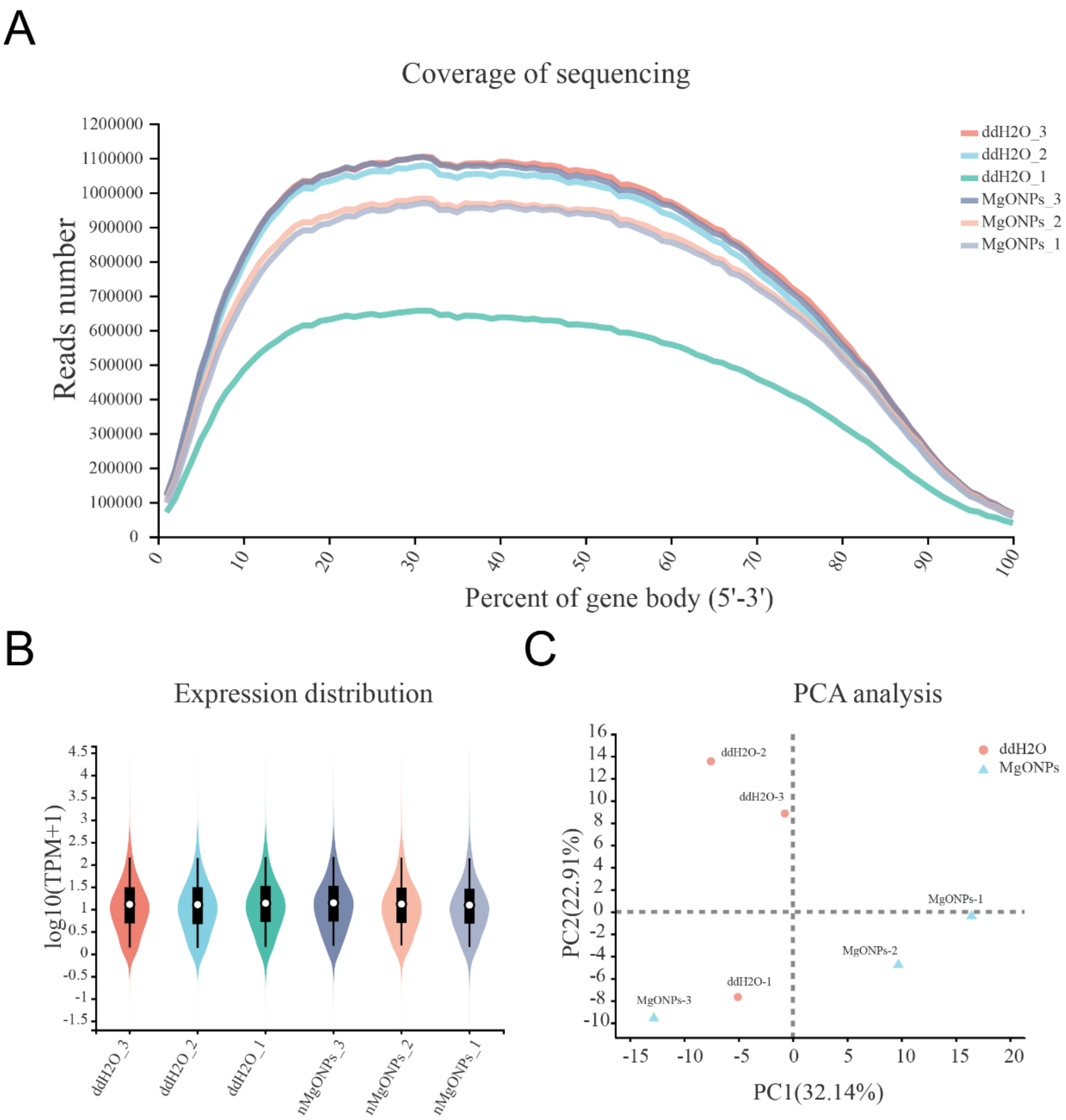

Figure S4. Coverage, expression distribution and principal component analysis (PCA) for different transcriptomes from potato. (A) Coverage of sequencing showed that our sequencing has no obvious bias. The X-axis represent the percentage of the base length in total base length of a single gene. "0" represents the 5′ end of the gene, "100" represents the 3′ end of the gene. The Y-axis showed that number of reads which can align to the corresponding gene region on the horizontal axis. The curve has no obvious deviation peak, so our sequencing has no obvious bias. (B) Boxplot showed that the distribution of all TPMs in each sample. The ordinate is log10(TPM+1), each color in the figure represents a sample, and the horizontal line in the figure represents the median level of gene expression in the sample. (C) The correlation analysis between different transcriptomes by principal component analysis (PCA).

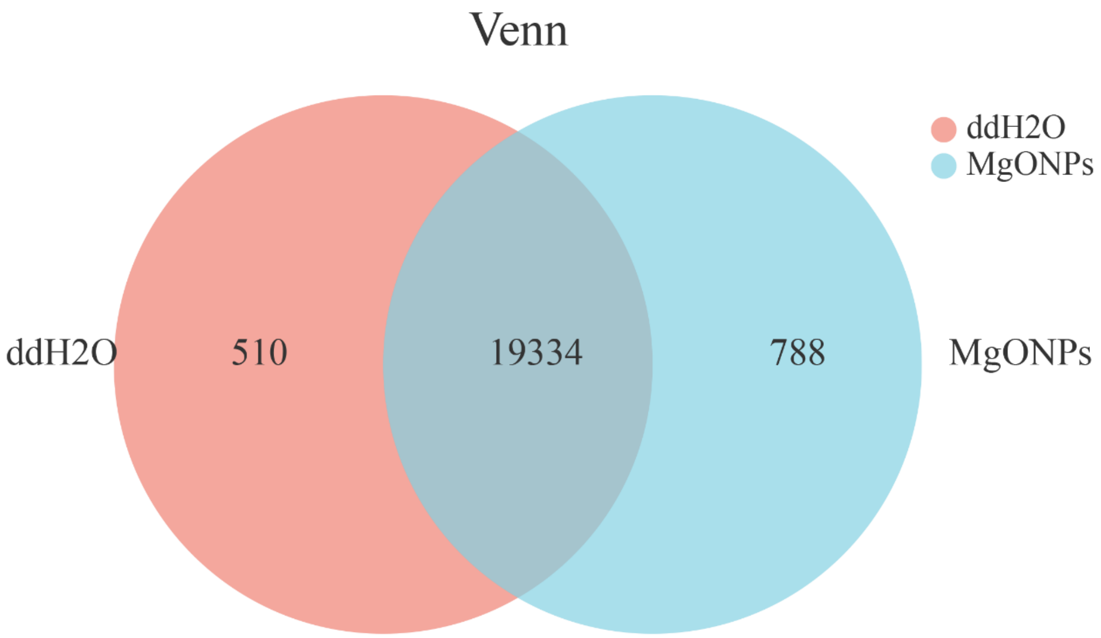

Figure S5. Venn diagram showing the number of expressing genes in potato upon MgONPs exposure and control.

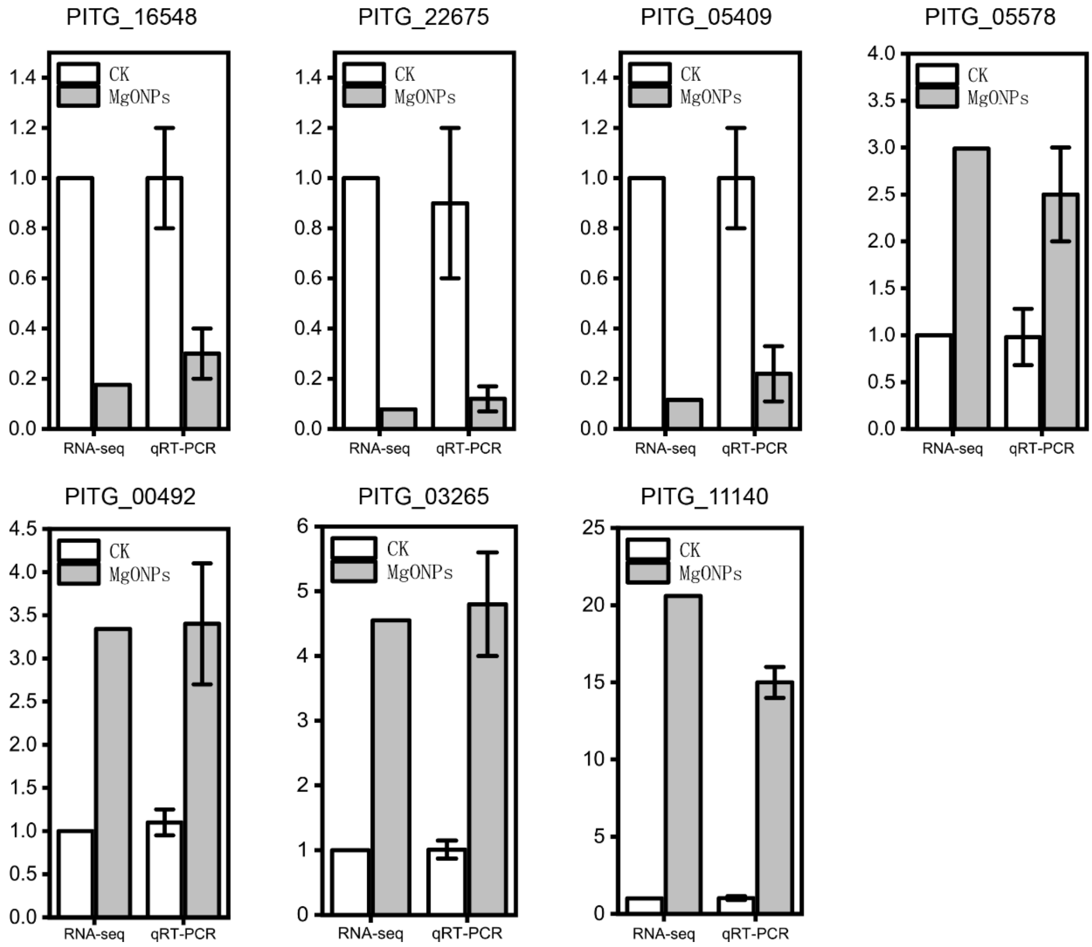

Figure S6. Comparison between transcriptome (RNA-seq data) and qRT-PCR data after sterile water (CK) or MgONPs exposure in *P. infestans*.

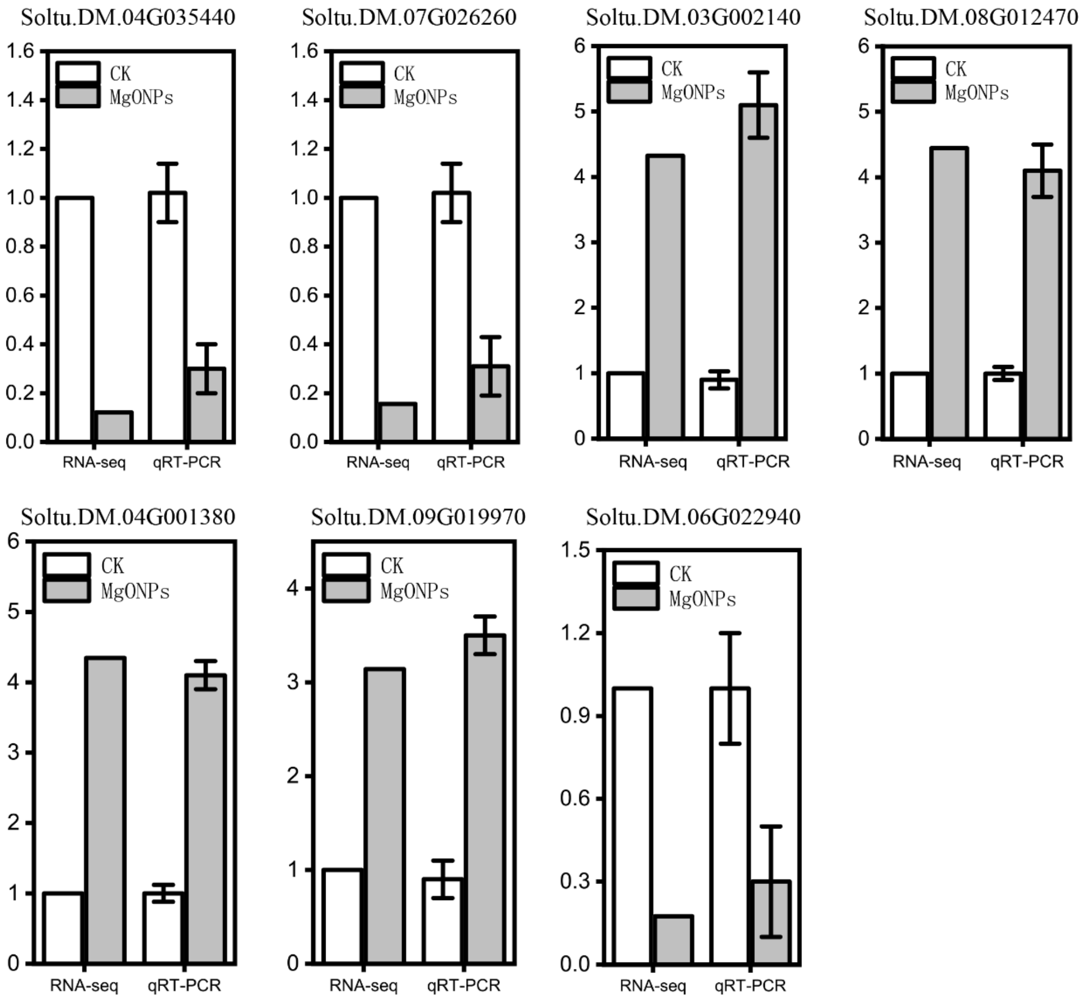

Figure S7. Comparison between transcriptome (RNA-seq data) and qRT-PCR data after sterile water (CK) or MgONPs exposure in potato leaves.

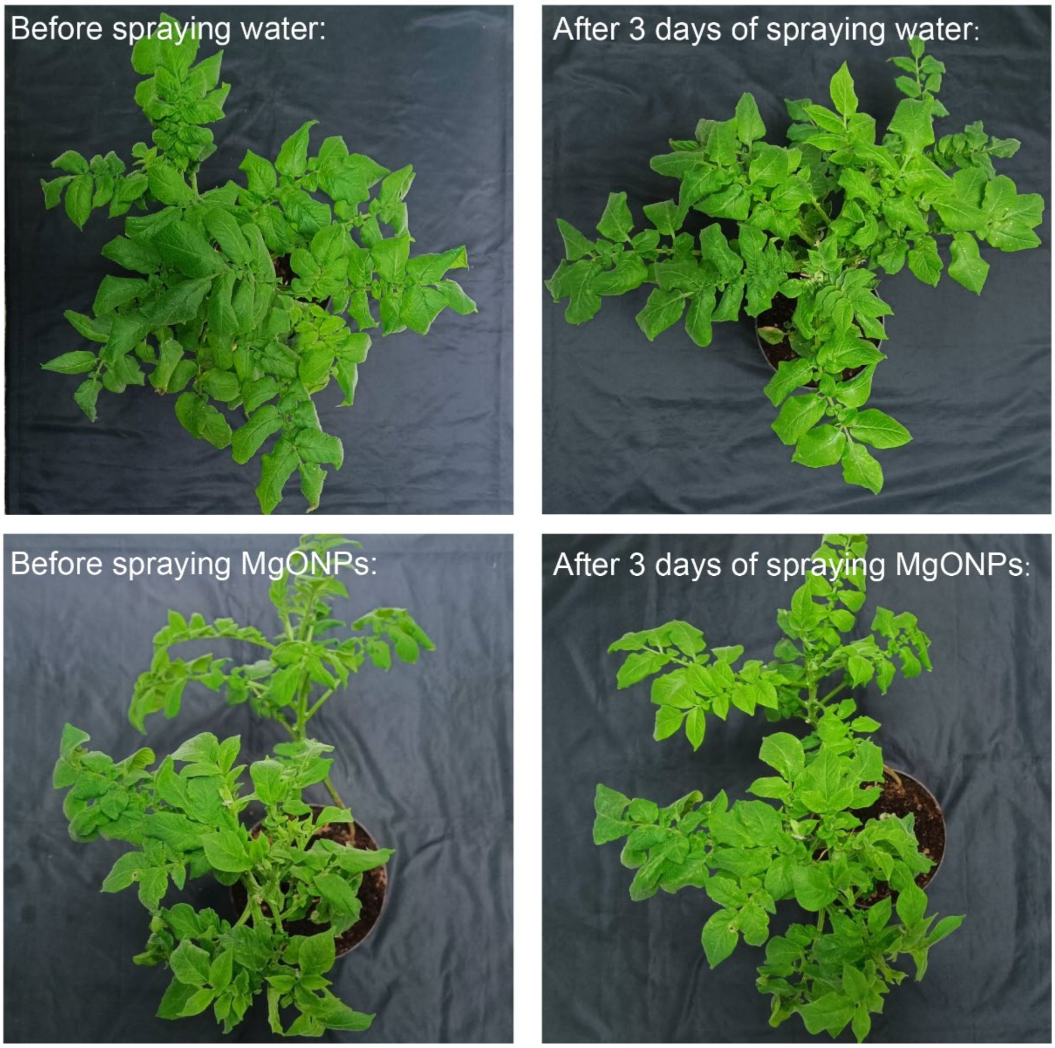

Figure S8. Photos of potatoes before MgONPs (250mg/L) or water treatment and after MgONPs (250mg/L) or water treatment 3 days.
